# STYK1 promotes tumor growth and metastasis by reducing SPINT2/HAI-2 expression in non-small cell lung cancer

**DOI:** 10.1038/s41419-019-1659-1

**Published:** 2019-06-04

**Authors:** Zhiqiang Ma, Dong Liu, Weimiao Li, Shouyin Di, Zhipei Zhang, Jiao Zhang, Liqun Xu, Kai Guo, Yifang Zhu, Jing Han, Xiaofei Li, Xiaolong Yan

**Affiliations:** 1 Department of Thoracic Surgery, Tangdu Hospital, The Fourth Military Medical University, 1 Xinsi Road, Xi’an, 710038 China; 2 State Key Laboratory of Cardiovascular Disease, Fuwai Hospital, National Center for Cardiovascular Diseases, Chinese Academy of Medical Sciences, Peking Union Medical College, 167 Beilishi Road, Beijing, 100037 China; 3 Department of Oncology, the Second Affiliated Hospital of Xi’an Jiaotong University, Xi’an, 710004 China; 4 Department of Aerospace Medicine, The Fourth Military Medical University, 169 Changle West Road, Xi’an, 710032 China; 5 Department of Ophthalmology, Tangdu Hospital, The Fourth Military Medical University, 1 Xinsi Road, Xi’an, 710038 China

**Keywords:** Non-small-cell lung cancer, Oncogenes

## Abstract

Non-small cell lung cancer (NSCLC) is the leading cause of cancer deaths worldwide. However, the molecular mechanisms underlying NSCLC progression remains not fully understood. In this study, 347 patients with complete clinicopathologic characteristics who underwent NSCLC surgery were recruited for the investigation. We verified that elevated serine threonine tyrosine kinase 1 (STYK1) or decreased serine peptidase inhibitor Kunitz type 2 (SPINT2/HAI-2) expression significantly correlated with poor prognosis, tumor invasion, and metastasis of NSCLC patients. STYK1 overexpression promoted NSCLC cells proliferation, migration, and invasion. STYK1 also induced epithelial–mesenchymal transition by E-cadherin downregulation and Snail upregulation. Moreover, RNA-seq, quantitative polymerase chain reaction (qRT-PCR), and western blot analyses confirmed that STYK1 overexpression significantly decreased the SPINT2 level in NSCLC cells, and SPINT2 overexpression obviously reversed STYK1-mediated NSCLC progression both in vitro and in vivo. Further survival analyses showed that NSCLC patients with high STYK1 level and low SPINT2 level had the worst prognosis and survival. These results indicated that STYK1 facilitated NSCLC progression via reducing SPINT2 expression. Therefore, targeting STYK1 and SPINT2 may be a novel therapeutic strategy for NSCLC.

## Introduction

Serine threonine tyrosine kinase 1 (STYK1), also known as NOK, was identified as a new member of the receptor protein tyrosine kinase (RPTK)-like protein family^1^. STYK1 shares 20–30% amino acids identity with the FGF/PDGF receptors, thus STYK1 belongs to a distinct member of these subfamily^1^. Like other RPTKs’ actions on promoting cancer progression^2^, STYK1 had been reported to promote cell proliferation of BaF3 cells and also induce rapid tumorigenesis and severe distant metastasis in nude mice^1^. Elevated STYK1 expression has been found in a wide range of cancers, including prostate cancer^3^, breast cancer^4^, liver cancer^5^, colorectal cancer^6^, acute leukemia^7^, and ovarian cancer^8^. Our previous study indicated that elevated STYK1 expression correlated with poor prognosis of non-small cell lung cancer (NSCLC) through using IHC analysis on 191 NSCLC patients^9^. However, whether STYK1 overexpression could promote NSCLC progression and the detailed mechanisms remain unclear.

Serine peptidase inhibitor Kunitz type 2 (SPINT2), also known as hepatocyte growth factor activator inhibitor 2 (HAI-2), is defined as a potent inhibitor of several serine proteases^10–12^. Numerous studies suggested SPINT2 acted as a tumor suppressor^13^, and inhibited cancer cell proliferation, metastasis, and invasion^10,11,14^. SPINT2 transcript is detectable in a variety of human tissues including lung^11^. Decreased SPINT2 expression has been found in several human cancers, such as liver cancer^15^, breast cancer^16^, prostate cancer^17^, ovarian cancer^18^, and cervical cancer^19^. Moreover, decreased SPINT2 level was also correlated with poor overall survival in these cancers^16–19^. However, the SPINT2 expression and its role in lung cancer are still unknown.

In the current study, we constructed the STYK1 overexpression (OE) NSCLC cell lines to investigate the actions of STYK1 on NSCLC progression both in vitro and in vivo. Our RNA-seq results showed the strong negative correlation between STYK1 and SPINT2 expression. Then we investigated whether SPINT2 involves in STYK1-mediated tumor progression. Furthermore, we assessed the expression of STYK1 and SPINT2 in 347 paired human NSCLC tissues and their correlation with clinicopathologic features and survival. We also analyzed whether the combined expressions of STYK1 and SPINT2 could serve as predictive markers for prognosis of NSCLC patients.

## Materials and methods

### Cell culture and lentivirus infection

Human NSCLC cell lines (H1299, Calu-1, SK-LU-1, H838, H322, A549, H157, SW-900 cells) and HEK-293T were obtained from the American Type Culture Collection (ATCC, VA, USA), and cultured in Dulbecco’s Modified Eagle Medium (DMEM) (Gibco, NY, USA), supplemented with 10% fetal bovine serum (FBS, Gibco), penicillin–streptomycin solution (100 units/ml) (Solarbio, Beijing, China). The STYK1, SPINT2, and empty vector lentiviruses were obtained from Genechem (Shanghai, China). Lentiviral infection in the H1299, Calu-1, and SK-LU-1 cells was conducted according to the protocol of the Genechem Recombinant Lentivirus Operation Manual provided by Genechem Corporation.

### NSCLC patient samples, tissue microarray, and immunohistochemistry (IHC)

Three-hundred and forty-seven NSCLC patients who underwent lung surgery at the Tangdu Hospital between May 2009 and January 2014 were included in this retrospective study under the approval of the ethics committee of the Fourth Military Medical University, and all the patients gave written informed consent on the use of clinical specimens for medical research. None of the patients had received preoperative chemotherapy or radiotherapy, and the complete follow-up was updated until death or January 2019, whichever came first. We randomly selected 24 pairs of frozen NSCLC tissues and corresponding adjacent noncancerous tissues for further western blot analysis. The 347 pairs of formalin-fixed NSCLC tissues and corresponding adjacent noncancerous tissues were made into paraffin-embedded tissue microarray. IHC staining was performed on tissue microarray sections using the primary antibodies of anti-STYK1 (1:50, ab97451, abcam) and anti-SPINT2 (1:200, HPA011101, Sigma), and the standard protocols were followed as previously described^9^. The scoring of the immunostaining degree was as 0 (negative), 1 (weak), 2 (moderate), and 3 (strong). Proportion of positive staining cells were scored as 0 (< 5%), 1 (6%–25%), 2 (26%–50%), 3 (51%–75%), and 4 (> 75%). These two scores were multiplied to produce total score. The NSCLC samples with low and high levels of STYK1 or SPINT2 expression were stratified by their respective average score.

### Analysis of cell viability

After cells seeded in 96-well plate, the cell viability was determined by the CCK-8 kit according to the manufacturer’s instructions (7Sea, Shanghai, China). Optical density (OD) values were obtained at 450 nm by the microplate reader (SpectraMax 190, Molecular Device, USA).

### Colony formation assay

Eight-hundred cells were seeded and cultured in the six-well plate for 12 days. Then, colonies were fixed with formalin and stained with 0.1% crystal violet (Solarbio, Beijing, China). After the plates were photographed, the colonies were solubilized by 30% acetic acid, and the absorbance was read at a wavelength of 540 nm according to previously described^20^.

### Transwell cell migration and invasion assay

For detecting the abilities of cell migration or invasion by transwell insert chambers, cells were seeded on the upper chamber with an uncoated or Matrigel-coated membrane (BD Biosciences). The upper chamber was filled with 300 μl DMEM medium without FBS, and the lower chamber was filled with 1000 μl DMEM containing 10% FBS. After 24 h, the migrated or invaded cells were fixed by formalin, stained by 0.1% crystal violet, photographed, and counted.

### Wound healing assay

Cells were seeded in the six-well plate in DMEM containing 10% FBS. Scratch was made by a 200 μl pipette tip after cells were grown to 90% confluence. After 24 h, the gaps between the wound edges were monitored and photographed under the microscope.

### RNA-seq and pathway enrichment analysis

Total RNA was extracted from NC and STYK1 OE groups of H1299 cells using TRIzol reagent (Invitrogen), and each group was prepared with three parallel replicates. Later, all the samples were sent to BGI Corporation (Shenzhen, China) for further RNA-seq detection and analysis via BGISEQ-500 sequencer. The pathway analysis for differentially expressed genes (DEGs) was performed based on the KEGG database. The data were analyzed on the Dr. Tom network platform of BGI (http://report.bgi.com).

### Real-time quantitative polymerase chain reaction (qRT-PCR)

The total RNA of NSCLC cells was extracted using TRIzol reagent, complementary DNA was generated using a Prime Script RT Master Mix. The primer sequences involved in qRT-PCR are as follows: SPINT2, forward AAGAATACTGCACCGCCAAC, reverse TTCTTCACCAGCTGCTCCTT; GAPDH, forward TGACTTCAACAGCGACACCCA, reverse CACCCTGTTGCTGTAGCCAAA. Later, qRT-PCR was conducted via the SYBR Premix Ex Taq II (TaKaRa, Dalian, China) to detect the targeted mRNAs levels. The GAPDH was set as the internal control.

### In vivo tumor xenograft assays

Male athymic nude mice were obtained from the Laboratory Animal Center of the Fourth Military Medical University. Different groups of 7 × 10^6^ H1299 cells were separately subcutaneously inoculated into the right flank of the nude mice for in vivo xenograft assay. The body weight and tumor size of each mouse were measured every 3 days for 21 days. Then tumors were excised from the sacrificed mice for additional analysis. The estimated tumor volume was calculated using the formula: volume = 0.5 × length × width^2^. All experimental procedures were approved by the Animal Ethics Committee of the Fourth Military Medical University, and in accordance with the ARRIVE (Animal Research: Reporting In Vivo Experiments) guidelines.

### Western blot and immunoprecipitation

Western blot procedures were presented as previously described^21^. As for immunoprecipitation, 500 μg lysates were incubated with the 3 μg anti-Flag agarose beads (A-2220, sigma) or the 3 μg anti-HA agarose beads (A-2095, sigma) at 4 °C for 4 h on a rotating incubator. Then immunocomplexes were washed four times with NETN buffer before being diluted with the loading buffer. The anti-STYK1 (1:1000, ab97451, abcam), anti-SPINT2 (1:1000, ab128926, abcam), anti-E-cadherin (1:1000, #14472, CST), anti-Snail (1:1000, #3879, CST), anti-β-actin (1:5000, ab6276, abcam), anti-tubulin (1:1000, #2148, CST), anti-Flag (1:2000, F-7425, sigma), anti-HA (1:1000, sc-805, santa cruz) were used as the primary antibodies. The 1:5000 dilution of the HRP-linked anti-IgG was used as the secondary antibody (Zhongshan Company, Beijing, China).

### 5-Ethynyl-2′-deoxyuridine (EdU) incorporation assay

Newly synthesized DNA in NSCLC cells was detected by the EdU fluorescence staining (C10638, Invitrogen) according to the manufacturer’s directions. Cells were visualized by the Olympus FV1000 confocal microscope (Olympus, Japan). The percentage of EdU-positive cells was shown as the ratio between the number of EdU-stained cells (red) and the total number of Hoechst 33342-stained cells (blue) counted × 100%.

### TUNEL assay

TUNEL in situ cell death detection kit (Beyotime, Shanghai, China) was used for detecting cellular apoptosis according to the manufacturer’s directions. Images were obtained by the confocal microscope (Olympus, Japan). The apoptotic index was calculated as the ratio between the number of TUNEL-stained nucleus (red) and the number of DAPI-stained nucleus (blue) counted × 100%.

### Statistical analyses

SPSS 23.0 (SPSS Inc., Chicago, USA) software was used to analyze the data. The *χ*^2^-test or Fisher’s exact test was used to assess the relationships between STYK1/SPINT2 expression and clinicopathological parameters of the NSCLC patients. Kaplan–Meier plots were used for overall survival rates, then compared with the logrank test. Univariate or multivariate survival analysis was carried out using the Cox proportional hazards model. Between two groups comparison were performed by Student’s *t*-test. Data are presented as the means ± SEM. Statistical significance was set at *P* < 0.05.

## Results

### Elevated expression of STYK1 in cancer tissues correlates with poor prognosis of NSCLC patients

**Fig. 1:**
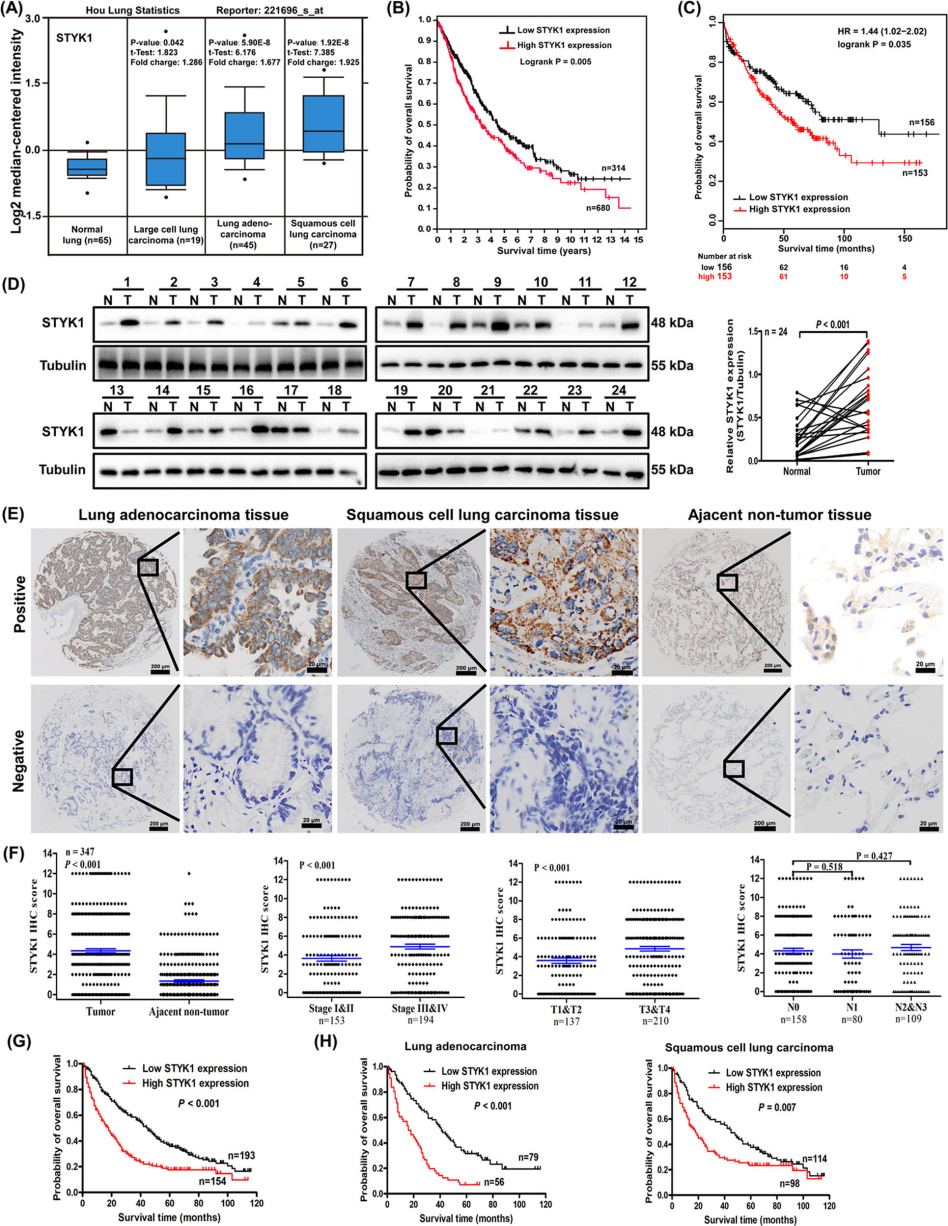
High STYK1 expression in cancer tissues was correlated with poor NSCLC prognosis. **a** The STYK1 mRNA levels of NSCLC compared with the normal lung sample in Hou lung Oncomine statistics. **b** Kaplan–Meier survival analysis about high/low STYK1 expression on 994 NSCLC patients based on The Human Protein Atlas (original RNA-seq data from TCGA), **c** on 309 NSCLC patients based on Kaplan–Meier plotter database. **d** Western blot analysis of STYK1 expression in tumor (T) and paired adjacent normal (N) tissues from 24 NSCLC patients. **e** Representative IHC images for STYK1 expression in NSCLC (LUAD and LUSC) and adjacent noncancerous tissues. Scale bar, 200 μm and 20 μm (inset), respectively. **f** Statistical analysis of STYK1 expression in 347 NSCLC patients through IHC staining. **g** Kaplan–Meier survival analysis about high/low STYK1 expression on 347 NSCLC patients and **h** 135 LUAD/212 LUSC patients based on our microarray tissue IHC results

To investigate the expression of STYK1 in NSCLC, we first performed data-mining to analyze the gene expression profiles of *STYK1* between cancer and normal tissues. Oncomine database (www.oncomine.org) showed that, the STYK1 mRNA levels were much higher in all the NSCLC subtypes (LCC, LUAD and LUSC) compared with the normal lung tissues (Fig. 1a). We next analyzed 994 NSCLC cases via Kaplan–Meier analysis in The Human Protein Atlas (the RNA-seq data based on The Cancer Genome Atlas, www.proteinatlas.org), and we found high STYK1 expression was related to poor prognosis (Fig. 1b). Interestingly, the Kaplan–Meier plotter database (http://kmplot.com) analysis also showed the positive correlation between high STYK1 expression and poor prognosis of NSCLC patients (HR = 1.44, Logrank *P* *=* 0.034, Fig. 1c).

We then measured the STYK1 expression in the initial cohort of 24 paired NSCLC and adjacent noncancerous tissues by western blot, and we found the STYK1 protein expression was significantly higher in tumor (T) than that in normal (N) tissues (Fig. 1d). To further confirm the STYK1 expression in NSCLC, the STYK1 protein level was detected by immunohistochemical analysis in tissue microarray containing 347 paired tumor-normal tissues (Fig. 1e). In all, 74.45% (258/347) NSCLC sections were classified as STYK1 positive while 47.84% (166/347) corresponding adjacent noncancerous tissue sections were classified as STYK1 positive. The STYK1 expression in NSCLC was significantly higher than that in the adjacent noncancerous samples (Fig. 1f). Moreover, NSCLC patients with deep tumor invasion (T3/T4) and high AJCC 8th stage (stages III/IV) had higher expression of STYK1 than these with superficial tumor invasion (T1/T2) and low AJCC 8th stage (stages I/II) (*P* < 0.001, Fig. 1f). The elevated STYK1 expression was positively correlated to tumor size, tumor invasion, distant metastasis, differentiation, and AJCC 8th stage (Table 1).

**Table 1:** Association of STYK1 and SPINT2 expression with clinicopathological parameters of patients with NSCLC

Category	*n*	STYK1 expression		*P-*value	SPINT2 expression		*P-*value
		Low	High		Low	High	
Age				0.41			0.806
<60	155	90	65		86	69	
≥ 60	192	103	89		104	88	
Gender				0.478			0.196
Male	278	152	126		157	121	
Female	69	41	28		33	36	
Tumor location				0.736			0.948
Left lung	143	78	65		78	65	
Right lung	204	115	89		112	92	
Tumor size				0.01			0.037
<5 cm	125	81	44		60	65	
≥ 5 cm	222	112	110		130	92	
Tumor invasion				<0.001			0.012
T1	6	2	4		2	4	
T2	131	92	39		61	70	
T3	130	71	59		72	58	
T4	80	28	52		55	25	
Lymphatic invasion				0.645			0.232
N0	158	90	68		81	77	
N1–N3	189	103	86		109	80	
Distant metastasis				0.011			0.002
No	337	191	145		179	157	
Yes	11	2	9		11	0	
Differentiation				0.003			<0.001
Well and moderate	238	145	93		110	128	
Poorly and not	109	48	61		80	29	
AJCC 8th stage				<0.001			<0.001
I	49	28	21		17	32	
II	104	75	29		48	56	
III	183	88	95		114	69	
IV	11	2	9		11	0	

Our Kaplan–Meier analysis results showed that NSCLC patients with high STYK1 expression were associated with worse overall survival (Logrank *P* *<* 0.001, Fig. 1g). Likewise, we got the similar conclusion of Kaplan–Meier analyses when the patients were divided into LUAD and LUSC groups (Fig. 1h). The 3- and 5-year cumulative survival rates (27.3% and 16.9%, respectively) for NSCLC patient with high STYK1 expression were much lower than these (58.5% and 36.8%, respectively) with low STYK1 expression (Table 2). After multivariate Cox survival analysis, STYK1 was found to be an independent prognostic factor for NSCLC patients (HR = 1.617, 95% CI: 1.254–2.084, *P* *<* 0.001, Table 3).

**Table 2:** Univariate analysis of the correlation between clinicopathological variables and survival of patients with NSCLC

Variables	Cumulative survival rates (%)		Mean survival time (mo)	Univariate analysis		
	3-Years	5-Years		HR	95% CI	*P-*value
Age				1.167	0.918–1.483	0.206
<60	50.3	29	40.75			
≥ 60	40.1	27.1	30.38			
Gender				1.078	0.798–1.456	0.626
Male	44.6	27.3	37.54			
Female	44.9	30.4	38.75			
Tumor location				0.886	0.696–1.127	0.323
Left lung	44.1	26.6	35.5			
Right lung	45.1	28.9	39.37			
Tumor size				1.606	1.245–2.073	<0.001
<5 cm	58.4	36	45.87			
≥ 5 cm	36.9	23.4	33.22			
Tumor invasion				2.051	1.591–2.644	<0.001
I–II	64.2	41.6	49.31			
III–IV	31.9	19	30.26			
Lymphatic invasion				1.761	1.378–2.251	<0.001
N0	57	38.6	46.77			
N1–N3	34.4	19	30.26			
Distant metastasis				2.386	1.302–4.373	0.005
No	45.5	28.9	38.42			
Yes	18.2	0	18.27			
Differentiation				5.219	3.977–6.849	<0.001
Well and moderate	60.1	40.3	48.85			
Poorly and not	11	0.9	13.61			
AJCC 8th stage				2.965	2.297–3.826	<0.001
I–II	69.9	47.7	54.4			
III–IV	24.7	12.4	24.64			
STYK1 expression				1.799	1.415–2.288	<0.001
Low	58.5	36.8	46.1			
High	27.3	16.9	27.34			
SPINT2 expression				0.429	0.334–0.551	<0.001
Low	29.5	13.7	27.77			
High	63.1	45.2	49.89

**Table 3:** Multivariate analysis of the correlation between clinicopathological variables of patients with NSCLC

Variables	Categories	Multivariate analysis			
		Standard error	HR	95% CI	*P*-value
Differentiation	Well and moderate/poorly and not	0.148	3.684	2.758–4.921	<0.001
AJCC 8th stages	I–II/III–IV	0.19	2.147	1.478–3.118	<0.001
STYK1 expression	Low/high	0.13	1.617	1.254–2.084	<0.001
SPINT2 expression	Low/high	0.14	0.569	0.433–0.748	<0.001

### STYK1 overexpression promotes NSCLC progression

**Fig. 2:**
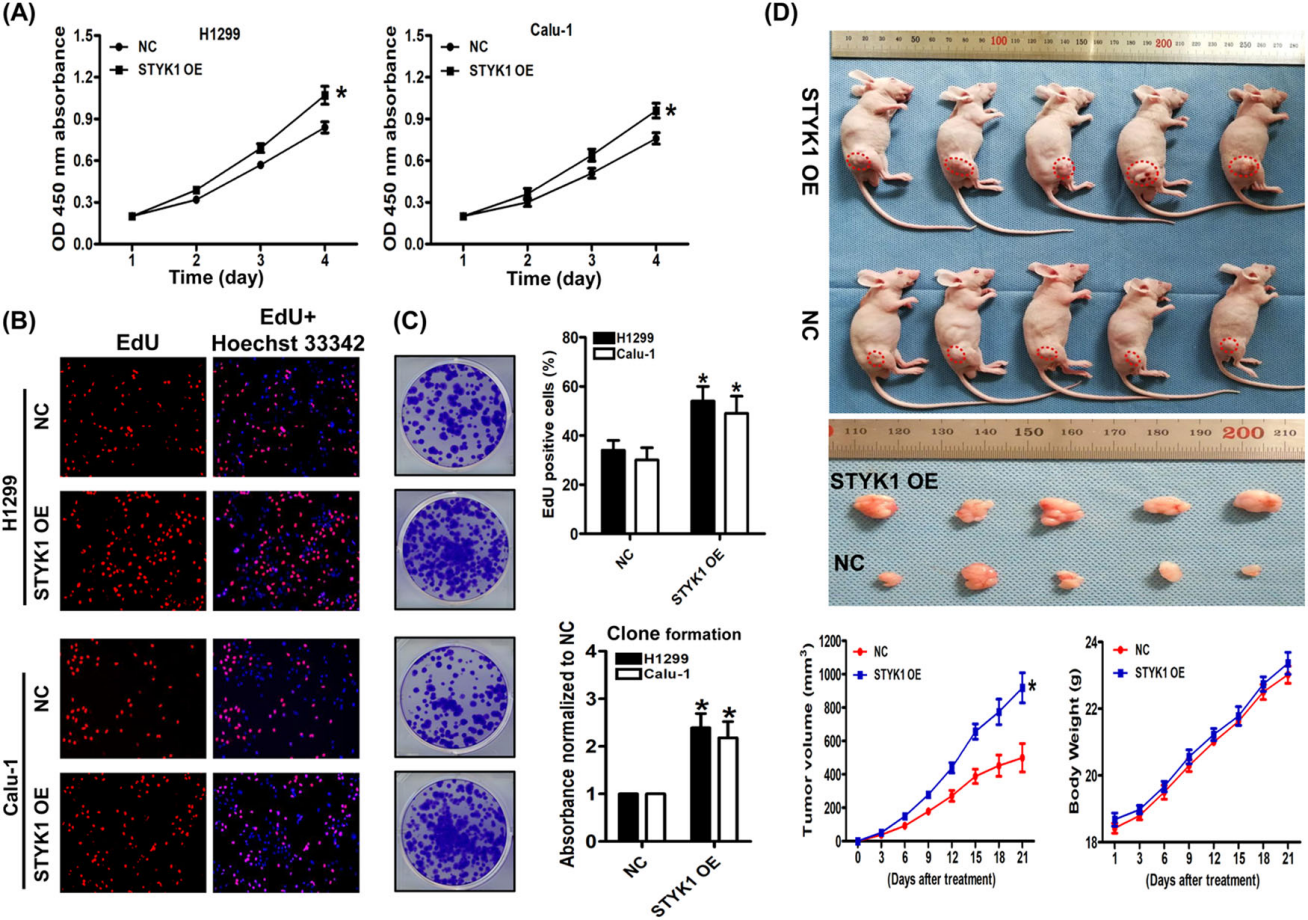
STYK1 overexpression facilitated NSCLC cells proliferation. **a** The growth curves of H1299 and Calu-1 cells. Cell viabilities were detected by CCK-8 assay and expressed as OD values. **b** Representative images and results of EdU incorporation assay. The result was calculated as the ratio between the number of EdU-stained cells (red fluorescence) and the total number of Hoechst 33342-stained cells (blue fluorescence). **c** The representative images and results of colony formation assay. The stained colonies were solubilized with 30% acetic acid; the absorbances were read at 540 nm and normalized to the NC group. **d** Photographs showing tumor xenograft morphologies in each group, changes in body weight of nude mice, and tumor growth curve drawn from the tumor volumes after subcutaneously injection of NC or STYK1 OE H1299 cells, respectively. ^*^P *<* 0.05 vs. the NC group

To study the role of STYK1 on NSCLC progression, we tested the STYK1 expression in multiple NSCLC cell lines (supplementary Fig. S1A), then we established the stable H1299 and Calu-1 STYK1 overexpression cell lines through using the STYK1 lentivirus. Compared with NC, STYK1 overexpression significantly increased the cell viability determined by CCK-8 analysis in both H1299 and Calu-1 cells (*P* *<* 0.05, Fig. 2a). We also used the EdU incorporation assay to analyze the role of STYK1 on proliferation, and we found STYK1 overexpression remarkably increased the EdU-positive cells compared with the NC group (*P* *<* 0.05, Fig. 2b). Moreover, this promoting effect was further validated by the enhanced colony formation ability in both cell lines with STYK1 OE compared with NC (*P* *<* 0.05, Fig. 2c). Interestingly, TUNEL assay showed that STYK1 overexpression had no effect on the basal apoptotic rate of NSCLC cells under normal cell culture (vs. NC group, *P* *>* 0.05, supplementary Fig. S1B). To further verify the action of STYK1 overexpression on promoting NSCLC cell proliferation, we then established H1299 cell xenograft in athymic nude mice and measured their tumor volumes. Consistent with the in vitro outcomes, we found that the mean volume of tumors of STYK1 OE group was 1.84-fold larger than that of NC group (Fig. 2d).

**Fig. 3:**
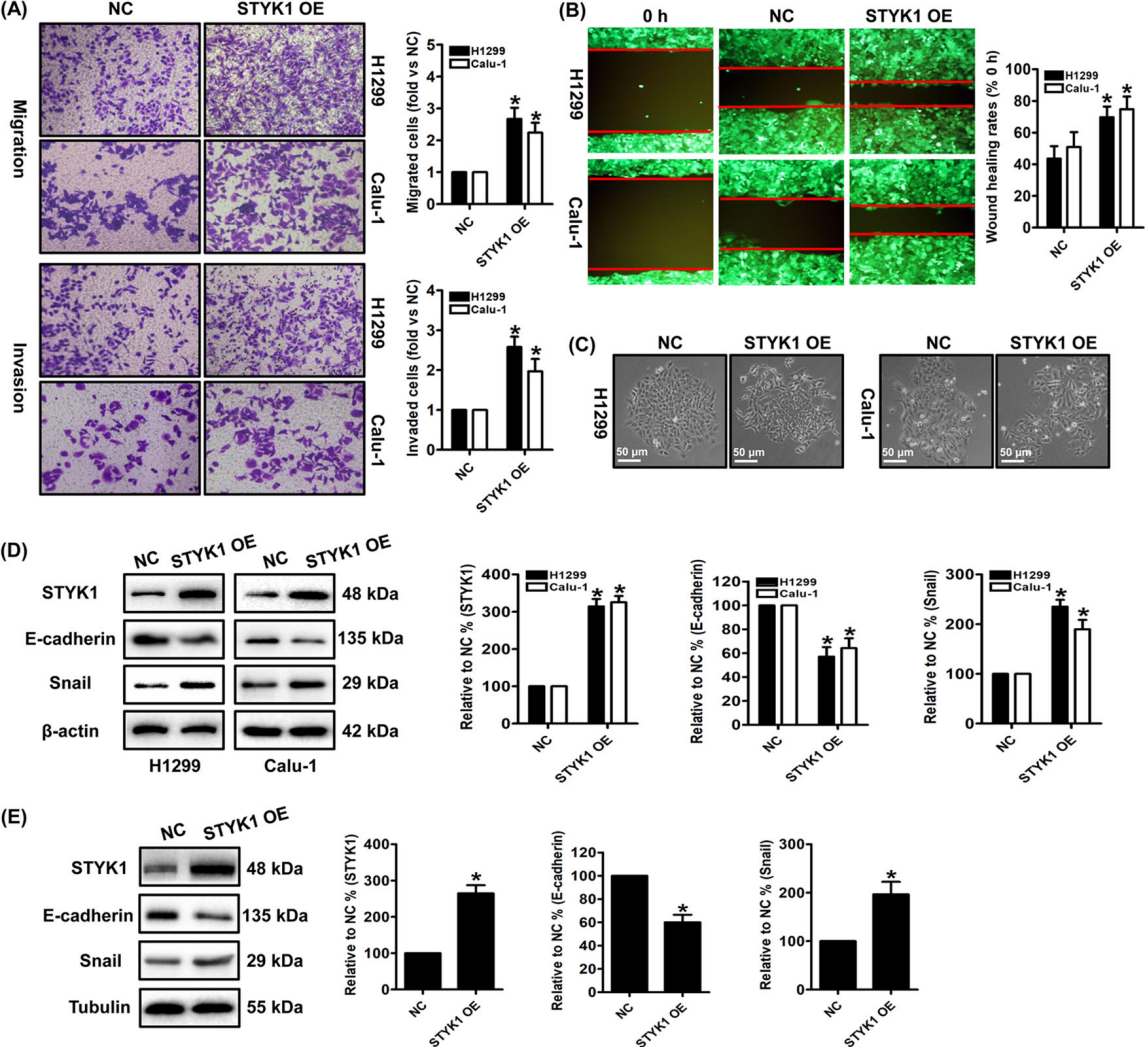
STYK1 overexpression facilitated NSCLC cells metastasis and invasion. **a** Representative images and results of transwell migration and invasion assay. **b** Representative wound healing images of each cells were shown, and the migratory ability is expressed as the mean distance between the two sides of the scratch. The initial scratched distance (at 0 h) was set as 100%. **c** STYK1 induces the mesenchymal morphology changes in H1299 and Calu-1 cells: spindle shape and loss of cell–cell contact. **d**, **e** Representative western blot results of STYK1, E-cadherin, Snail in **d** H1299 and Calu-1 cells and in **e** H1299 xenograft tumor tissues were shown. Membranes were re-probed for β-actin or Tubulin expression to show that similar amounts of protein were loaded in each lane. ^*^P *<* 0.05 vs. the NC group

Moreover, the migratory and invasive potentials were also enhanced by STYK1 overexpression in both H1299 and Calu-1 cells. These actions were multi-validated by transwell migration and invasion assays, and the wound healing assay (Fig. 3a, b). To further detect the role of STYK1 on NSCLC cell lines from the primary sites, we then overexpressed the STYK1 in SK-LU-1 cells and found STYK1 overexpression also enhanced the migratory and invasive abilities in SK-LU-1 cells (supplementary Fig. S2A). Interestingly, after the NSCLC cells infected with STYK1 lentivirus, we observed spindle shape change in NSCLC cells, and the cells lost cell–cell contact and were scattered in some cell colonies (Fig. 3c). These phenomena suggested that the STYK1 overexpression promoted the NSCLC cells’ epithelial–mesenchymal transition (EMT) potential, which plays important roles on cancer metastasis. We then measured the EMT biomarkers E-cadherin and Snail expression by western blot, and we found STYK1 overexpression significantly decreased anti-EMT E-cadherin expression and increased pro-EMT Snail levels both in vitro and in vivo (Fig. 3d, e).

### SPINT2 is the downstream target of STYK1 in NSCLC

**Fig. 4:**
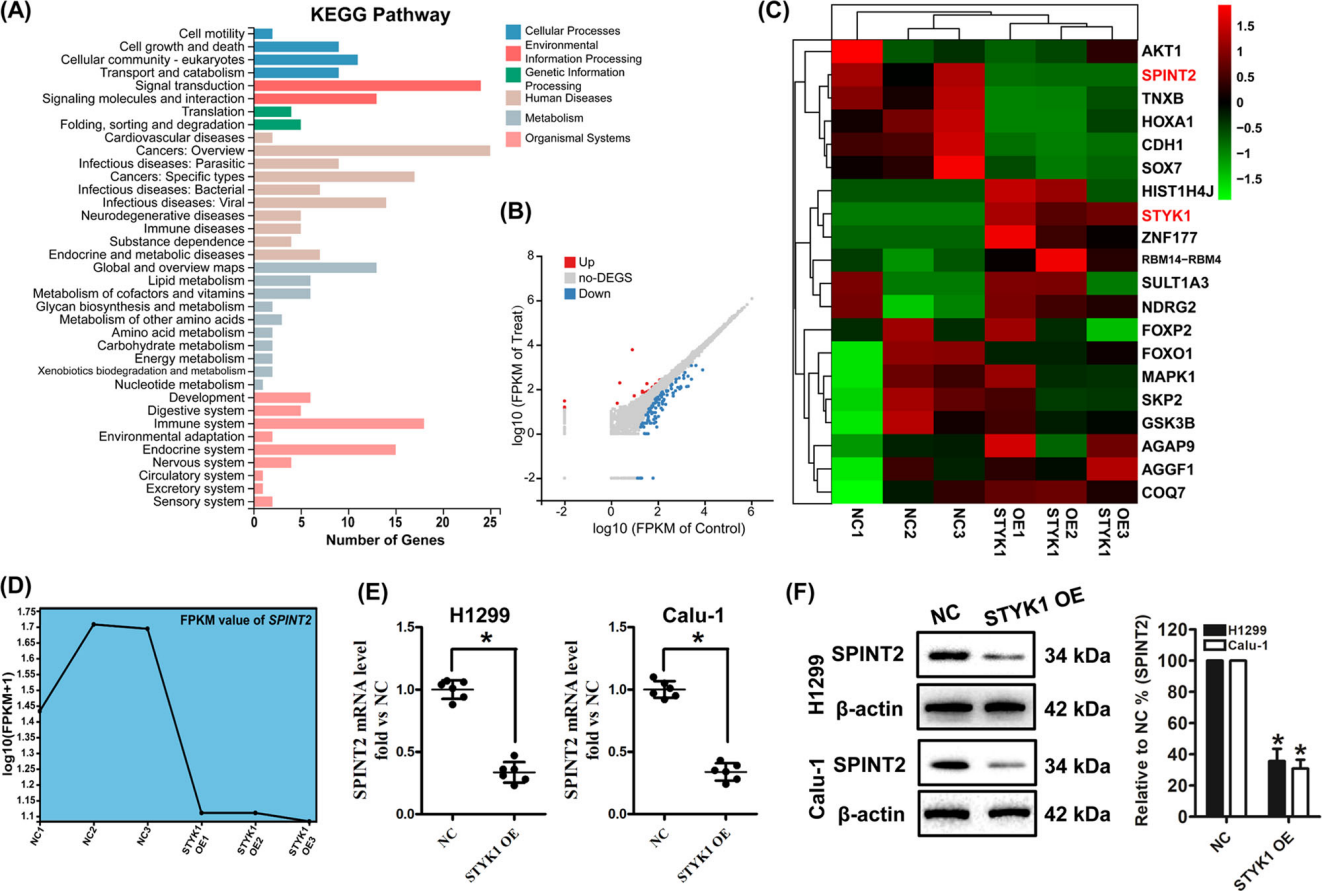
SPINT2 level was significantly downregulated by STYK1 overexpression both in vivo and in vitro. **a** KEGG pathway analysis based on the RNA-seq results for NC vs. STYK1 OE in H1299 cells. *P-*value was corrected by FDR, and the FDR < 0.01 was considered to be significantly enriched. **b** Representative Scatter Plot of 134 significant genes (20 upregulated genes marked in red and 114 downregulated genes marked in blue) for NC vs. STYK1 OE. **c** Representative heatmap of gene expression levels. **d** Representative FPKM value of SPINT2 in three replicates of the NC and STYK1 OE groups. **e** mRNA levels of SPINT2 measured by qRT-PCR were shown. **f** Representative western blot result of SPINT2 levels. Membranes were re-probed for β-actin expression to show that similar amounts of protein were loaded in each lane. ^*^P *<* 0.05 vs. the NC group

To further explore the underlying molecular mechanisms of STYK1 overexpression on promoting NSCLC progression, the RNA-seq based transcriptome analysis to be used as estimate the transcriptome changes in H1299 cells among the NC and STYK1 OE groups. There were 20 genes upregulated and 114 genes downregulated in the STYK1 OE group compared with the NC group (Fig. 4a, b). KEGG pathway analysis showed that these genes were related to cellular motility, growth and death, etc. Among these genes, we focused on *SPINT2*, whose expression was strongly downregulated by STYK1 overexpression (Fig. 4c, d). Previous studies suggested SPINT2 acts as an anticancer molecular and inhibits cancer development and progression^11,22^. Decrease of SPINT2 mRNA level was further validated by qRT-PCR analysis in the STYK1 OE group compared with NC group in both H1299 and Calu-1 cells (*P* < 0.05, Fig. 4e). Moreover, western blot analysis also revealed that SPINT2 protein level was significantly downregulated in the STYK1 OE NSCLC cells (Fig. 4f).

### SPINT2 involves in STYK1-mediated NSCLC progression

**Fig. 5:**
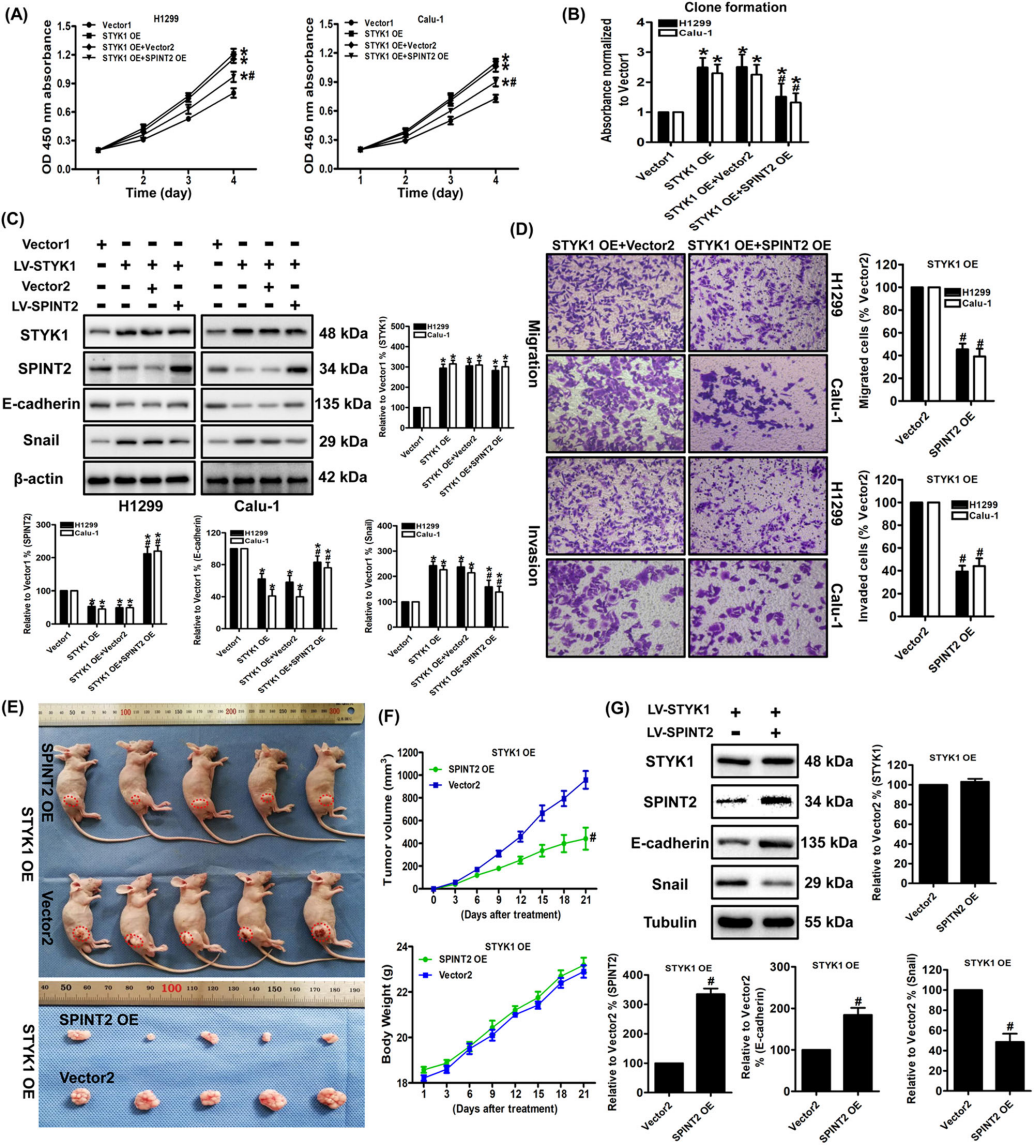
SPINT2 was essential for STYK1-mediated NSCLC cell proliferation, migration, and invasion. **a** The growth curves of H1299 and Calu-1 cells. Cell viabilities were detected by CCK-8 assay and expressed as OD values. **b** The representative results of colony formation assay. Stained colonies were solubilized with 30% acetic acid; the absorbances were read at 540 nm and normalized to the Vector1 group. **c** Representative western blot results of STYK1, SPINT2, E-cadherin, Snail were shown. Membranes were re-probed for β-actin expression to show that similar amounts of protein were loaded in each lane. **d** Representative images and results of transwell migration and invasion assay. **e** Photographs showing tumor xenograft morphologies in each group, **f** changes in body weight of nude mice, and tumor growth curve drawn from the tumor volumes after subcutaneously injection of STYK1 OE + Vector2 and STYK1 OE + SPINT2 OE H1299 cells, respectively. **g** Representative western blot results of STYK1, SPITN2, E-cadherin, Snail were shown. Membranes were re-probed for Tubulin expression to show that similar amounts of protein were loaded in each lane. ^*^P *<* 0.05 vs. the Vector1 group, ^#^*P* *<* 0.05 vs. the STYK1 OE + Vector2 group

To confirm the involvement of SPINT2 in the STYK1-mediated NSCLC progression, we examined the actions of SPINT2 upregulation by infecting SPINT2 lentivirus on the H1299 and Calu-1 cells overexpressing STYK1. The expression of SPINT2 was verified by western blot, and SPINT2 overexpression had no effect on STYK1 levels (Fig. 5c). Moreover, the immunoprecipitation results suggested that STYK1 did not bind to SPINT2 at the protein level in both HEK-293T and H1299 cells with upregulated STYK1 and SPINT2 expression (Supplementary Fig. S2B, C). After detecting the cell viability and using the colony formation assay, we found SPINT2 overexpression partially inhibited the enhanced proliferative ability in both H1299 and Calu-1 cells overexpressing STYK1 (Fig. 5a, b). Furthermore, the in vivo study showed that SPINT2 overexpression significantly decreased the mean volume of tumors in the H1299-STYK1 OE xenograft group (Fig. 5e, f). Previous studies reported that STYK1 promotes Akt phosphorylation in cancer cells^23,24^, and we also found STYK1 overexpression could increase the p-Akt levels in NSCLC cells (Supplementary Fig. S2D). Interestingly, SPINT2 overexpression markedly reversed STYK1 OE-induced Akt phosphorylation in NSCLC cells (Supplementary Fig. S2D). Moreover, the STYK1-enhanced migratory and invasive abilities were weakened by SPINT2 overexpression through analyzing the transwell migration and invasion assays (Fig. 5d). SPINT2 overexpression also reversed the STYK1 OE-induced EMT marker’s changes by increasing E-cadherin and decreasing Snail levels both in vitro and in vivo (Fig. 5c–g). Therefore, the above results indicated that STYK1-mediated NSCLC progression can be reversed by SPINT2 upregulation.

### Correlation of STYK1 and SPINT2 expression with NSCLC prognosis

**Fig. 6:**
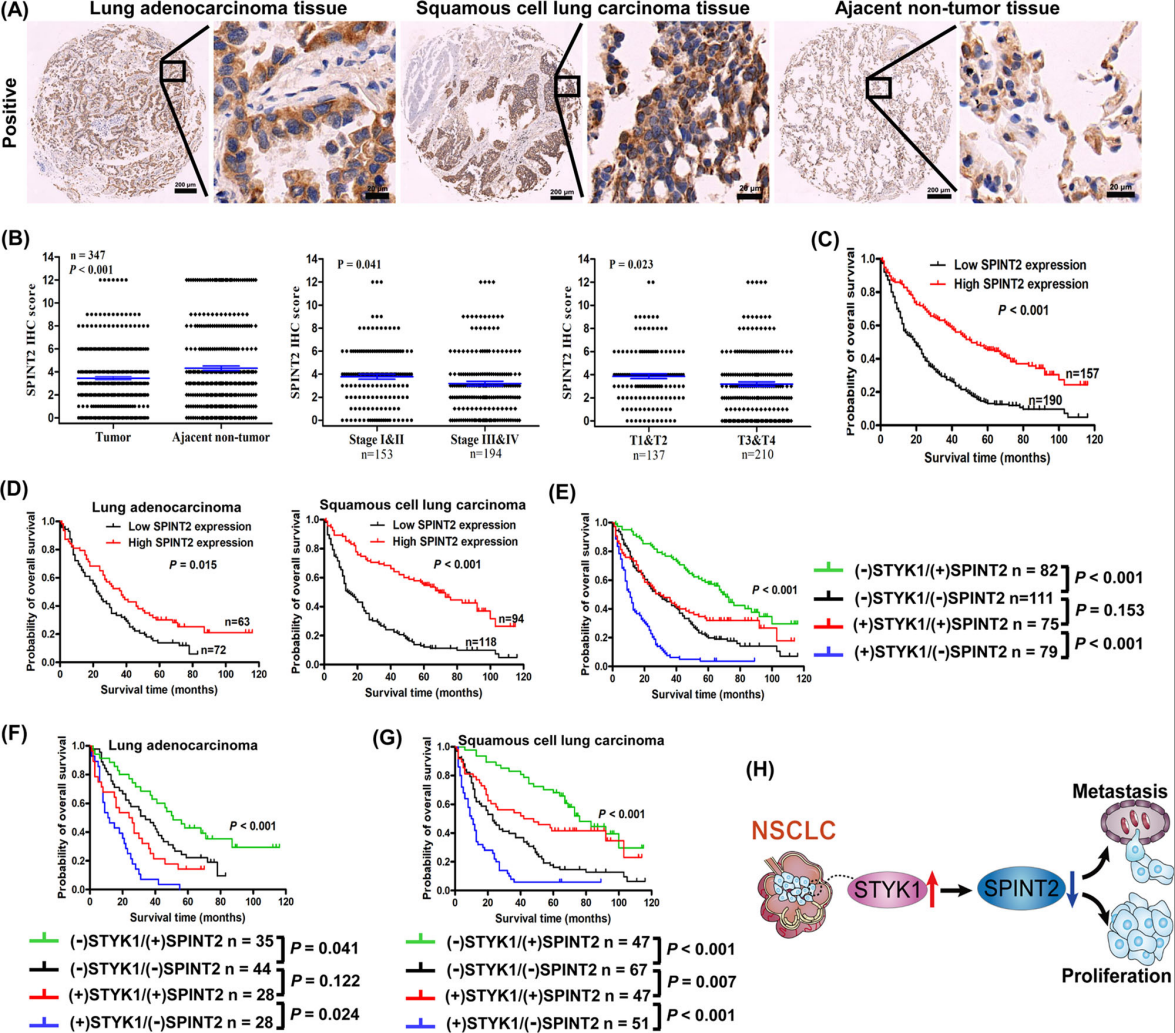
Correlation between STYK1 and SPINT2 expression in NSCLC tissue samples. **a** Representative IHC images for SPINT2 expression in NSCLC (LUAD and LUSC) and adjacent noncancerous tissues. Scale bar, 200 μm and 20 μm (inset), respectively. **b** Kaplan–Meier survival analysis about high/low SPINT2 expression on 347 NSCLC patients and **d** 135 LUAD/212 LUSC patients based on our microarray tissue IHC results. **c** Statistical analysis of SPINT2 expression in 347 NSCLC patients through IHC staining. **e** Kaplan–Meier analysis of the association between overall survival and the expression of STYK1 and SPINT2 on 347 NSCLC patients, **f** 135 LUAD patients, and **g** 212 LUSC patients based on our microarray tissue IHC results. **h** Schematic diagram about the decrease of SPINT2 involving in the elevated STYK1-mediated NSCLC progression

After verifying the relation between STYK1 and its downstream target SPINT2, we further detected the SPINT2 expression by IHC tissue array analysis containing 347 paired tumor-normal samples (Fig. 6a). The SPINT2 expression in NSCLC was much lower than that in the adjacent noncancerous samples (*P* *<* 0.001, Fig. 6b). Moreover, NSCLC patients with deep tumor invasion (T3/T4) and high AJCC 8th stage (stages III/IV) had significantly lower expression of SPINT2 than these with superficial tumor invasion (T1/T2) and low AJCC 8th stage (stages I/II) (Fig. 6b). The high SPINT2 expression was negatively correlated to tumor invasion, distant metastasis, differentiation, and AJCC 8th stage (Table 1). Furthermore, Kaplan–Meier survival curves indicated that NSCLC patients with high SPINT2 expression were associated with better prognosis (Fig. 6c); similar outcomes were also found in the stratified LUAD and LUSC subgroups (Fig. 6d). The 3- and 5-year cumulative survival rates (63.1% and 45.2%, respectively) for NSCLC with high SPINT2 expression were much higher than NSCLC (29.5% and 13.7%, respectively) with low SPINT2 expression (Table 2). Moreover, the multivariate Cox survival analysis indicated that SPINT2 expression was positively correlated with NSCLC overall survival (HR = 0.569, 95% CI: 0.433–0.748, *P* *<* 0.001, Table 3).

Next, we set out to detect whether prediction of NSCLC prognosis was more accurate according to combined STYK1 and SPINT2 expression than themselves alone. Patients were divided into four groups: low STYK1/high SPINT2, low STYK1/low SPINT2, high STYK1/high SPINT2, and high STYK1/low SPINT2 groups. After analyzing the total NSCLC group and the stratified LUAD and LUSC groups, Kaplan–Meier survival curves indicated that patients with low STYK1/high SPINT2 had the best prognosis, and patients with high STYK1/low SPINT2 had the worst prognosis (Fig. 6e–g). In both high and low STYK1 level subgroups, patients with high SPITN2 level showed better prognosis than the low one (Fig. 6e–g). Interestingly, in the NSCLC group and the LUAD subgroup, there was no statistically difference of patients’ survival between the low STYK1/low SPINT2 group with high STYK1/high SPINT2 group (*P* > 0.05, Fig. 6e, f); whereas LUSC with high STYK1/high SPINT2 showed better prognosis than LUSC with low STYK1/low SPINT2 (*P* *=* 0.007, Fig. 6g).

## Discussion

NSCLC accounts for the most cases of lung cancer, which is the leading cause of cancer deaths worldwide^25–27^. There is an urgent need to further understand the molecular mechanisms of NSCLC progression and identify new prognostic markers and therapeutic targets^28–31^. In this study, our databases-mining results indicated that STYK1 mRNA levels were much higher in the NSCLC compared with the normal lung tissues, and high STYK1 expression was related to poor NSCLC prognosis. Furthermore, the above outcomes were confirmed by our IHC analysis results based on tissue microarray containing 347 paired tumor-normal NSCLC samples. In addition, multivariate Cox survival analysis indicated STYK1 was an independent prognostic factor for NSCLC patients. Chen et al.^9^ indicated that elevated STYK1 expression was a predictor of poor prognosis in LUSC but not in LUAD patients. They discussed that these outcomes still warrant further validation due to fewer investigated cases. Interestingly, our results based on larger cohort cases suggested that high STYK1 level was related to worse survival of both LUSC and LUAD patients.

Several studies reported that STYK1 overexpression could promote cancer cell proliferation^1,5,8^, but the action of STYK1 on NSCLC cell proliferation has not been previously verified. In this study, we found the proliferative ability (cell viability and colony formation) was significantly enhanced by STYK1 overexpression in NSCLC cells. Consistently, the IHC analysis results indicated that elevated STYK1 expression was positively correlated to NSCLC tumor size and tumor invasion. Additionally, the promotion of tumor growth by STYK1 was further confirmed in subcutaneous xenograft tumor nude mice model.

EMT is viewed as a critical intermediate step in tumorigenesis and plays critical role in cancer metastasis^24,32,33^. STYK1 has been previously reported as a potent EMT inducer and promotes several cancer metastasis such as liver cancer^5^, cervical cancer^24^, and gallbladder cancer^34^. After overexpressing STYK1 in NSCLC cells, we observed EMT-related spindle shape change in H1299 and Calu-1 cells, and the cells lost cell–cell contact and were scattered in some cell colonies. Later, western blot analysis suggested that STYK1 overexpression significantly decreased anti-EMT E-cadherin expression and increased pro-EMT Snail levels both in vitro and in vivo. These results indicated EMT may be induced by STYK1 overexpression in NSCLC cells. Our IHC analysis results found that the elevated STYK1 expression was positively correlated to distant metastasis, differentiation, and AJCC 8th stage. Moreover, we also found the migratory and invasive potentials of NSCLC cells were significantly enhanced by STYK1 overexpression.

*SPINT2* gene is a putative tumor suppressor, and encodes a transmembrane protein with two extracellular Kunitz domains that inhibits a variety of serine proteases, such as hepatocyte growth factor activator, trypsin, plasmin, kallikreins, and hepsin^35^. SPINT2 protein level has been proposed as a marker of favorable prognosis by its suppressive actions on cancer cell growth, EMT, metastasis, and invasion^10,14^. SPITN2 transcript is detectable in a variety of human tissues including lung^11^. Our RNA-seq results indicated the *SPINT2* gene expression was significantly decreased in the H1299 cells overexpressing STYK1. This result was further validated by our qRT-PCR and western blot analyses both in H1299 and Calu-1 cells. Interestingly, SPINT2 overexpression had no effect on STYK1 levels, and SPINT2 overexpression significantly reversed the STYK1-enhanced proliferative, migratory, and invasive abilities both in vitro and in vivo. Moreover, the expression of EMT markers (E-cadherin and Snail) were also revered after SPINT2 upregulation in NSCLC cell overexpressing STYK1. Akt is one of the most frequently activated molecules in human cancers^36–39^. Consistent with previous studies^23,24^, we found STYK1 overexpression could increase the Akt phosphorylation in NSCLC cells. SPINT2 upregulation was reported to decrease p-Akt levels in melanoma cells^11,40^. Interestingly, we found SPINT2 overexpression markedly reversed STYK1 OE-induced Akt phosphorylation in NSCLC cells. The above results indicated that SPINT2 was the downstream target of SYTK1 and involved in STYK1-mediated NSCLC progression.

To our best of knowledge, SPINT2 has not been reported as an independent prognostic factor in NSCLC. In current study, we also detected the SPINT2 expression by IHC tissue array analysis. We found the SPINT2 expression in NSCLC was much lower than that in the adjacent noncancerous samples. Decreased SPINT2 expression was positively correlated to tumor invasion, distant metastasis, differentiation, and AJCC 8th stage. Moreover, we found low SPINT2 expression strongly correlated with worse overall survival in NSCLC, LUAD, and LUSC patients. These patients with low SPINT2 expression had a significantly shorter overall survival time than those with high expression. When combining STYK1 and SPINT2 expression for further analyses, Kaplan–Meier survival curves showed that patients with high STYK1/low SPINT2 had the worst prognosis in NSCLC, LUAD and LUSC. This result also suggested that elevated STYK1 and decreased SPINT2 promoted NSCLC progression. Interestingly, LUAD with low STYK1/low SPINT2 showed no statistically difference of patients’ survival compared with high STYK1/high SPINT2 group; whereas LUSC with high STYK1/high SPINT2 showed better prognosis than LUSC with low STYK1/low SPINT2. These results indicated that the expression and function of STYK1 and SPITN2 may be affected and regulated by other potential signaling cascades and mechanisms in different NSCLC subtypes, which warrants further investigation.

Taken together, our studies verified that STYK1 functioned as a tumor promoting factor through enhancing NSCLC cell growth and metastasis. Moreover, we found elevated STYK1 or decreased SPINT2 expression strongly correlated with NSCLC poor prognosis, and downregulation of SPINT2 involved in STYK1-mediated NSCLC progression (Fig. 6h). Therefore, targeting STYK1 and SPINT2 could be a promising therapeutic strategy for future therapies of NSCLC.

## Supplementary information

Supplementary Fig. S1

Supplementary Fig. S2
