# Supplementary material for: STYK1 promotes tumor growth and metastasis by reducing SPINT2/HAI-2 expression in non-small cell lung cancer
Source: Cell Death Dis. 2019 Jun 4;10(6):435. doi: 10.1038/s41419-019-1659-1 (PMC6547759; doi:10.1038/s41419-019-1659-1)
Supplement: Supplementary file 1 — Supplementary Fig. S1 [file 41419_2019_1659_MOESM1_ESM.docx]

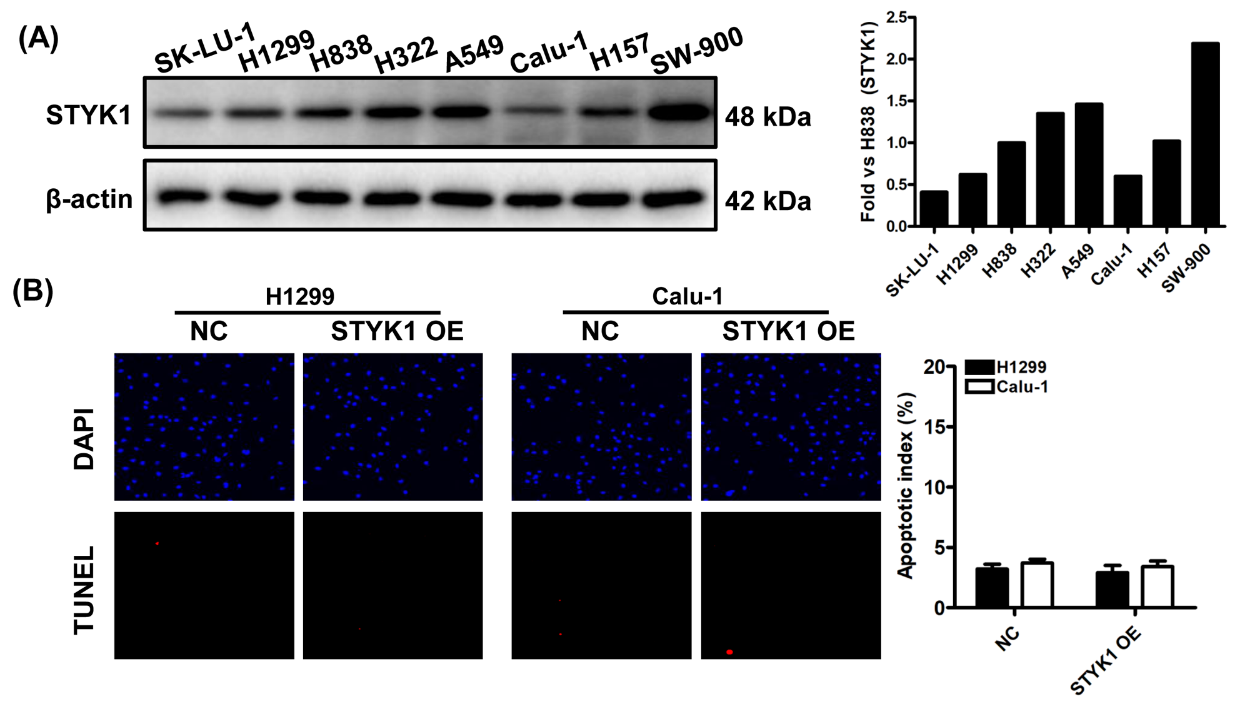


**Supplementary Fig. S1** (A) Representative western blot results of STYK1 expression in NSCLC cell lines. Membranes were re-probed for β-actin expression to show that similar amounts of protein were loaded in each lane. The STYK1 expression in H838 cells was set as 1.0. (B) Representative images of TUNEL staining were obtained and the basal apoptotic rates in H1299 and Calu-1 cells were calculated and presented.
