# Supplementary material for: STYK1 promotes tumor growth and metastasis by reducing SPINT2/HAI-2 expression in non-small cell lung cancer
Source: Cell Death Dis. 2019 Jun 4;10(6):435. doi: 10.1038/s41419-019-1659-1 (PMC6547759; doi:10.1038/s41419-019-1659-1)
Supplement: Supplementary file 2 — Supplementary Fig. S2 [file 41419_2019_1659_MOESM2_ESM.docx]

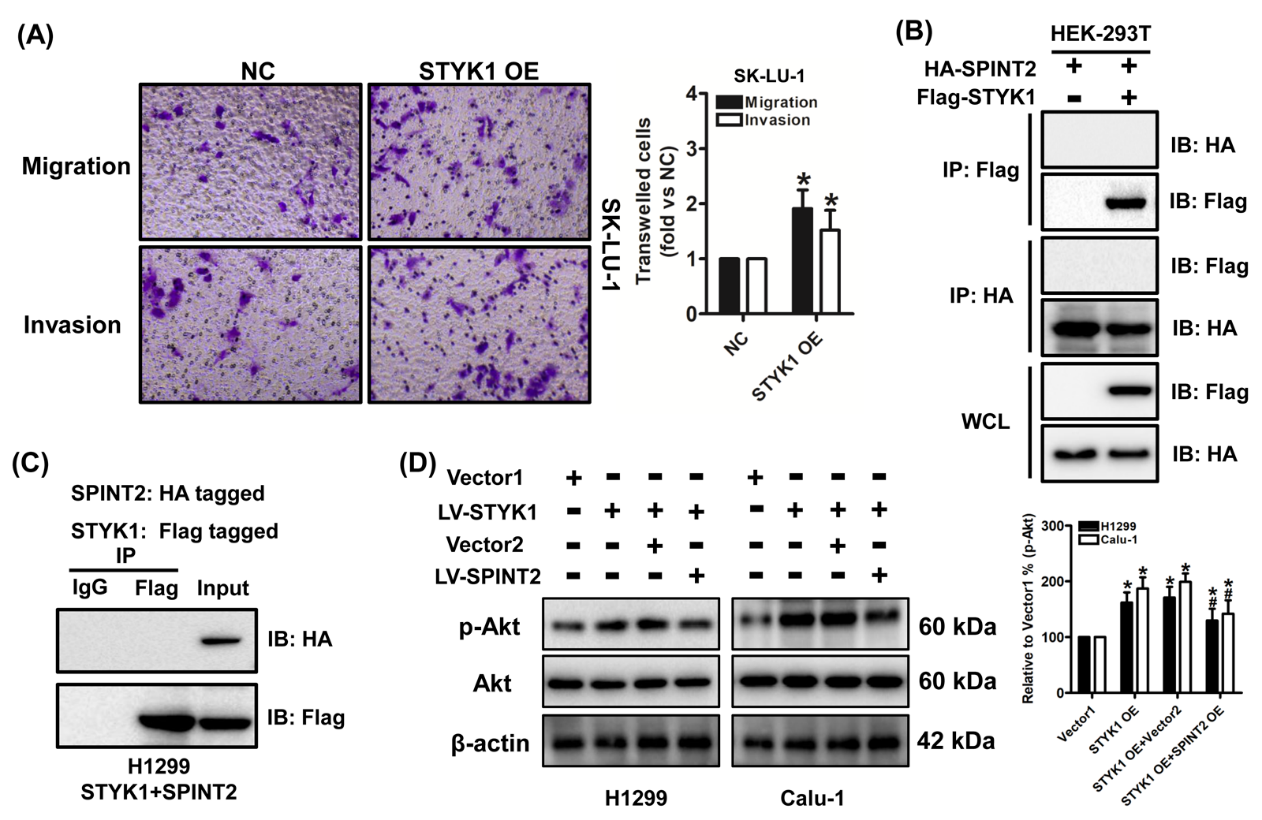


**Supplementary Fig. S2** (A) Representative images and results of transwell migration and invasion assay in SK-LU-1 cells. ^*^*P* < 0.05 *vs*. the NC group. (B) and (C), STYK1 did not bind to SPINT2 at the protein level in cells. (B) Immunoblot (IB) analysis of immunoprecipitation (IP) and whole cell lysates (WCL) derived from HEK-293T cells transfected with HA-SPINT2 with the Flag-STYK1 construct, and (C) the Immunoblot analysis of immunoprecipitation derived from H1299 cells transfected with HA-SPINT2 with the Flag-STYK1 construct. (D) Representative western blot results of p-Akt and Akt were shown. Membranes were re-probed for β-actin expression to show that similar amounts of protein were loaded in each lane. ^*^*P* < 0.05 *vs*. the Vector1 group, ^#^*P* < 0.05 *vs*. the STYK1 OE+Vector2 group.
